# A phylogenetic approach to study the evolution of somatic mutational processes in cancer

**DOI:** 10.1038/s42003-022-03560-0

**Published:** 2022-06-22

**Authors:** Sayaka Miura, Tracy Vu, Jiyeong Choi, Jeffrey P. Townsend, Sajjad Karim, Sudhir Kumar

**Affiliations:** 1 Institute for Genomics and Evolutionary Medicine, Temple University, Philadelphia, PA USA; 2 Department of Biology, Temple University, Philadelphia, PA USA; 3 Program in Computational Biology and Bioinformatics, Yale University, New Haven, CT USA; 4 Department of Biostatistics, Yale School of Public Health, New Haven, CT USA; 5 Yale Cancer Center, Yale University, New Haven, CT USA; 6 Center for Excellence in Genomic Medicine Research, King Abdulaziz University, Jeddah, Saudi Arabia

**Keywords:** Statistical methods, Tumour heterogeneity, Molecular evolution

## Abstract

Cancer cell genomes change continuously due to mutations, and mutational processes change over time in patients, leaving dynamic signatures in the accumulated genomic variation in tumors. Many computational methods detect the relative activities of known mutation signatures. However, these methods may produce erroneous signatures when applied to individual branches in cancer cell phylogenies. Here, we show that the inference of branch-specific mutational signatures can be improved through a joint analysis of the collections of mutations mapped on proximal branches of the cancer cell phylogeny. This approach reduces the false-positive discovery rate of branch-specific signatures and can sometimes detect faint signatures. An analysis of empirical data from 61 lung cancer patients supports trends based on computer-simulated datasets for which the correct signatures are known. In lung cancer somatic variation, we detect a decreasing trend of smoking-related mutational processes over time and an increasing influence of APOBEC mutational processes as the tumor evolution progresses. These analyses also reveal patterns of conservation and divergence of mutational processes in cell lineages within patients.

## Abstract (web summary)

A method, PhyloSignare, is presented to infer mutational signatures along a tumour evolutionary tree and is used to analyse somatic genomic variations in 61 lung cancer patients, providing insights into tumour evolution.

## Introduction

**Fig. 1:**
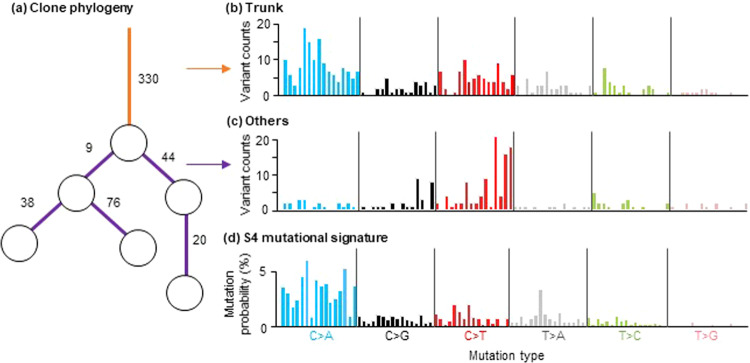
Clone phylogeny and variant counts from a lung cancer patient. **a** Clone phylogeny of 6 clones. Clones are shown with circles. Numbers along branches represent variant counts. **b**, **c** Observed variant counts in the trunk (orange branch; **b**) and the other branches (purple; **c**). The data were obtained from Jamal-Hanjani et al. (2017) (CRUK0025 dataset). **d** COSMIC signature S4 is characterized by many C to A mutations.

Tumor cells accumulate somatic mutations during cancer progression marked by dynamic demography of cells, including emergence, expansion, and extinction^1,2^. Researchers now routinely reconstruct mutational histories and clone phylogenies by analyzing genome sequence variation^3–7^. These variants can be localized to individual branches in a clone phylogeny and relative frequencies of different variant types may be compared across branches to detect shifts in cellular mutational processes over time (Fig. 1). For example, C → A transversions are more frequent in the trunk of a clone phylogeny than in its descendants in the clone phylogeny in Fig. 1, suggesting that mutagenic processes have changed over time in this lung cancer patient^8^. Inference of such changes in mutational processes will enhance our understanding of the intricacies of tumor evolution, including the effect of pre-existing genetic alterations, behavioral changes, and treatment regimens that lead to changes in mutational processes^9–14^.

Change in mutational processes is detected by analyzing their outcomes: types of variants and their relative counts. For example—unless tissue sample preparation has induced C → A changes due to oxidation—a large C → A variant frequency is a tell-tale sign of smoking-related mutational processes (COSMIC signature S4; Fig. 1b, d). Smoking-related mutations decline after smoking cessation^15,16^ (Fig. 1c). In contrast, age-related mutagenic processes create C → T transitions that arise across the human lifespan (COSMIC signature S1), particularly at methylated CpG sites^17–20^. Many such distinct mutational signatures have been identified from extensive and comprehensive large-scale data analysis of the tumor genetic variation in different cancers and have been assembled in online catalogs^20,21^. For example, 30 signatures have been recognized in COSMIC version 2, each a vector of 96 different mutational contexts consisting of the mutated base and adjacent 5′ and 3′ bases (e.g., Fig. 1d)^20,22,23^.

Generally, shifts of mutational processes during tumor evolution have been identified by contrasting dominant signatures detected from variants in primary tumors and those from metastatic tumors or by comparing early (clonal) and late (subclonal) variants^9–14^. Researchers have also begun to analyze branch-specific mutational signatures in clone phylogenies to discover mutagens and variants linked with the origin of new clones in cancer patients^10,24–26^. Many computational methods are available to estimate relative activities of mutational signatures for a given collection of genetic variants and their frequencies, such as quadratic programming (QP), deconstructSigs, MutationalPatterns, and sigLASSO^27–30^.

**Fig. 2:**
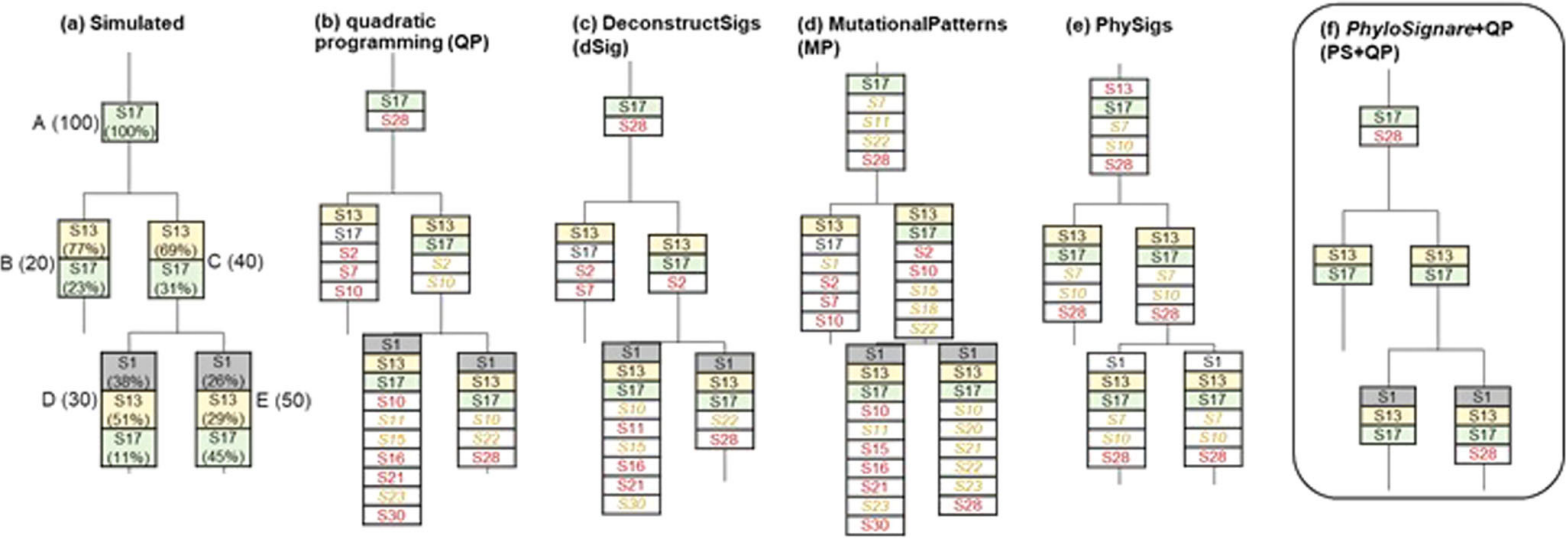
Mutational signatures detected by different methods for individual branches in the simulated clone phylogeny. **a** Model clone phylogeny and simulated mutational signatures. There are five branches: A – E with 20–100 variants (counts in parentheses next to the branch name) and each signature’s relative activity (shown below the signature name). See *Methods* for the detail. **b**–**e** Mutational signatures inferred by using different methods: **b** Quadratic programming [QP], **c** DeconstructSigs [dSig], and (**d**) MutationalPatterns [MP]. These methods QP, dSigs, and MP are expected to show limited accuracy due to a small number of variants. **e** Mutational signatures are inferred using PhySigs (optimal solution), which consider the evolution of mutational signatures along clone phylogenies. **f** Mutational signatures are inferred by applying the *PhyloSignare* approach with the QP Method (PS + QP). The number of incorrect signatures detected became smaller after coupling QP with *PhyloSignare*. Incorrectly detected signatures are shown with red (>5% estimated relative activity) and yellow italic (0.1%–5% estimated relative activity) letters, and correct signatures not detected are shown in white boxes with black letters.

The refitting methods were originally developed to detect mutational signatures for tumor samples, but they may be applied to the collection of variants mapped onto individual branches in the clone phylogeny, e.g., Fig. 1a. However, the direct application of refitting methods to infer branch-specific variants may produce many spurious signatures, while other correct signatures remain undetected (Fig. 2; Supplementary Figs. S1 and S2). The difficulty seems to be greater for branches with the fewest number of variants (Fig. 2). Similar issues were seen when we used methods that assume linear clonal evolution (CloneSig^31^) and PhySigs^32^ that detect mutational signature shifts in the given clone phylogeny (Fig. 2e**;** Supplementary Fig. S2c). Such errors hamper reliable detection of branch-specific signatures and inference of their evolution in a patient, limiting us to gross comparisons^11,25,33,34^.

We hypothesized that the performance of refitting methods (e.g., QP) in estimating relative activities of a given collection of signatures could be improved through a joint analysis of collections of variants mapped on proximal branches of the clone phylogeny. This is because neighboring branches in the clone phylogeny are expected to share some mutational signatures due to their shared environment and evolutionary history (e.g., Dentro et al.^12^). Therefore, we developed an approach called *PhyloSignare* (PS) to leverage the proximity of evolutionary lineages in the clone phylogeny to improve the performance of refitting methods in detecting mutational signatures.

In the following, we present PS and evaluate its accuracy by analyzing computer-simulated datasets. We compared the performance of the phylogeny-aware application of refitting methods (e.g., PS + QP) with PhySigs, which directly uses clone phylogeny and detects mutational signature shifts on branches. Finally, we applied PS to infer mutational signature evolution in non-small cell lung cancer patients, revealing branch-specific mutational signatures at a finer phylogenetic resolution.

## Results

The key distinguishing feature of our approach to applying existing refitting methods is that PS aims to reduce the complexity of signature detection for a given branch in the phylogeny. This is done by pooling variants from proximate relatives of the focused branch and then applying refitting methods to detect candidate signatures. Then the importance of each candidate signature is estimated by assessing the impact of exclusion of a signature on the fit of the signature and its activity. Thus, PS does not use relative activity as the only way to select signatures, preventing the detection of spurious signatures.

### The *PhyloSignare* (PS) approach

**Fig. 3:**
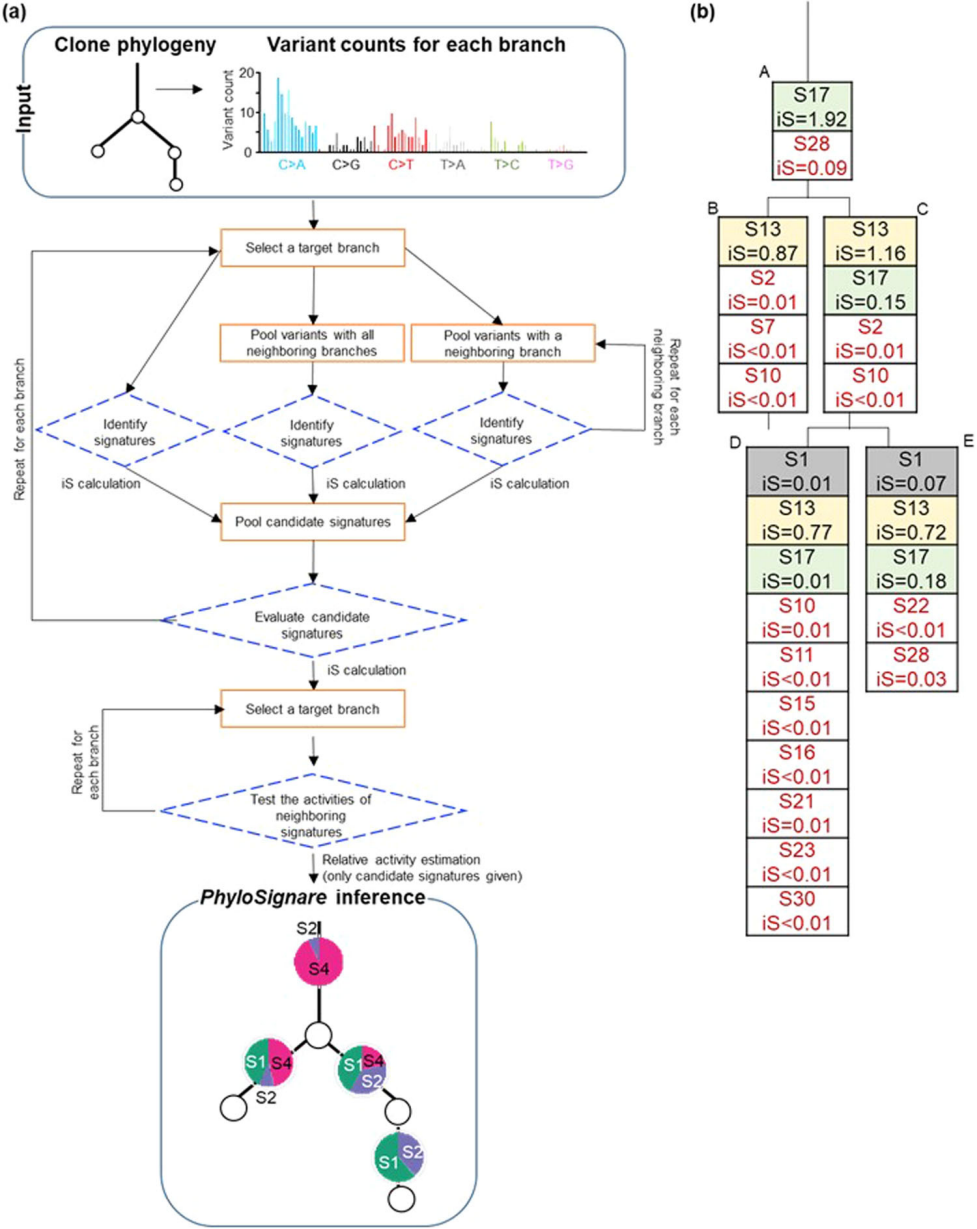
Overview of PhyloSignare approach. **a** Our approach uses a clone phylogeny in which all variants are mapped along branches. *PhyloSignare* pools mutations with adjacent branches and collect candidate signatures for each branch. We use iS statistics (see text) to evaluate the presence of each candidate signature. Last, we test if signatures from neighboring branches are active at a branch. Signatures will be detected for each branch. **b** We used variants for each branch and detected signatures by using QP. Detected signatures and iS are shown in a box. Signatures with red letters are incorrect detection, and iS values were relatively small.

Figure 3a shows a flowchart of the PS approach. The input is a clone phylogeny and variant counts for each branch (Fig. 3a), and the output is a set of signatures and their relative activity for every branch (see *Methods* for details). PS applies a refitting method (e.g., QP) to estimate the relative activities of mutational signatures for the observed variant counts, followed by the estimation of an importance score (iS) for every inferred signature. iS contrasts the fit of the predicted signatures to explain the frequencies of branch-specific variants with and without the given signature (Eq. 1; see *Methods* section for details). When iS is small, the predicted signature may be spurious. For example, iS2, iS7, and iS10 for signatures S2, S7, and S10, respectively, were small (<0.02) in the analysis of variants mapped to branch B in the computer-simulated dataset (Figs. 2a, 3b). None of these signatures were simulated on this branch (Fig. 2a). In contrast, the correct signature S13 received a high score (0.87). Therefore, iS is a simple heuristic to find spurious candidate signatures in a branch-by-branch analysis with the potential to reduce the false-positive detection of signatures. We used a simple cut-off of iS = 0.02 determined by observed iS for correct and incorrect signatures for the 180 simulated phylogenies (see *Methods* section) (Supplementary Fig. S3a). One may also use a chi-square test instead of a fixed iS cut-off, but the chi-square test becomes powerless when the number of variants is small.

Returning to our example, we found that QP did not detect one correct signature, S17, on branch B (Fig. 2b). The pooling of variants in branch B with its ancestral branch (trunk, branch A) identified S17 as a candidate signature. S17 was also detected when variants on branch B were pooled with its other neighbors. This happens because pooling variants from neighboring branches in the clone phylogeny increases the number of variants in the collection, helping the refitting method. Analyzing pooled variants for branch B, we identified six candidate signatures: S2, S7, S10, S13, S17, and S28. Next, the PS approach uses iS to evaluate each candidate signature to retain only the most reliable signatures for a branch. We have a set of candidate signatures for every branch at this stage.

In the final step, PS seeks the most economical gain and loss signature scenario in the phylogeny by expanding the collection of candidate signatures for each branch to include the signatures in its neighbors. For example, collecting candidate signatures on branch B now includes S17 because its immediate relatives A and C have that as a candidate signature. Then, the refitting method estimates the relative activities of all the candidate signatures branch-by-branch. The estimated activity of every candidate signature is then reported.

PS + QP removed most of the incorrect signatures originally detected by using QP alone (Fig. 2f). Also, the sum of iS values for all signatures (Overall_iS) detected on a branch improved by 2%−56% in PS + QP as compared to QP alone, which suggests that the inclusion of spurious signatures results in a poorer fit.

### Improvement of accuracy after coupling with PhyloSignare (PS)

We tested the improvement of accuracy after coupling with PS using 180 datasets that were previously simulated^32^. These multi-clone phylogenies contained five or seven branches, with fewer than 100 variants mapping to 486 branches out of 1080 (see *Methods*). Signatures were randomly sampled from 30 COSMIC signatures (v2) to select a set of signatures for a clone phylogeny. In a phylogeny, loss (too low to be detected) and/or gain of signatures were introduced up to two times. We assessed the performance of PS coupled with three refitting methods: QP, deconstructSigs, and MutationalPatterns (PS + QP, PS + dSig, and PS + MP, respectively). We did not couple PS with sigLASSO because we found sigLASSO to have a high false-negative rate, which resulted in a lack of a sufficient number of signatures available for analysis with PS (Supplementary Fig. S2b). We also did not couple PS with CloneSig (and related methods) and PhySigs because these methods already consider clone phylogeny. Instead, we compared the performance of PS-based approaches with PhySigs. CloneSig, the best among related methods that assume a linear phylogeny, was not pursued further because linear clone phylogenies are not common^8,35^.

**Fig. 4:**
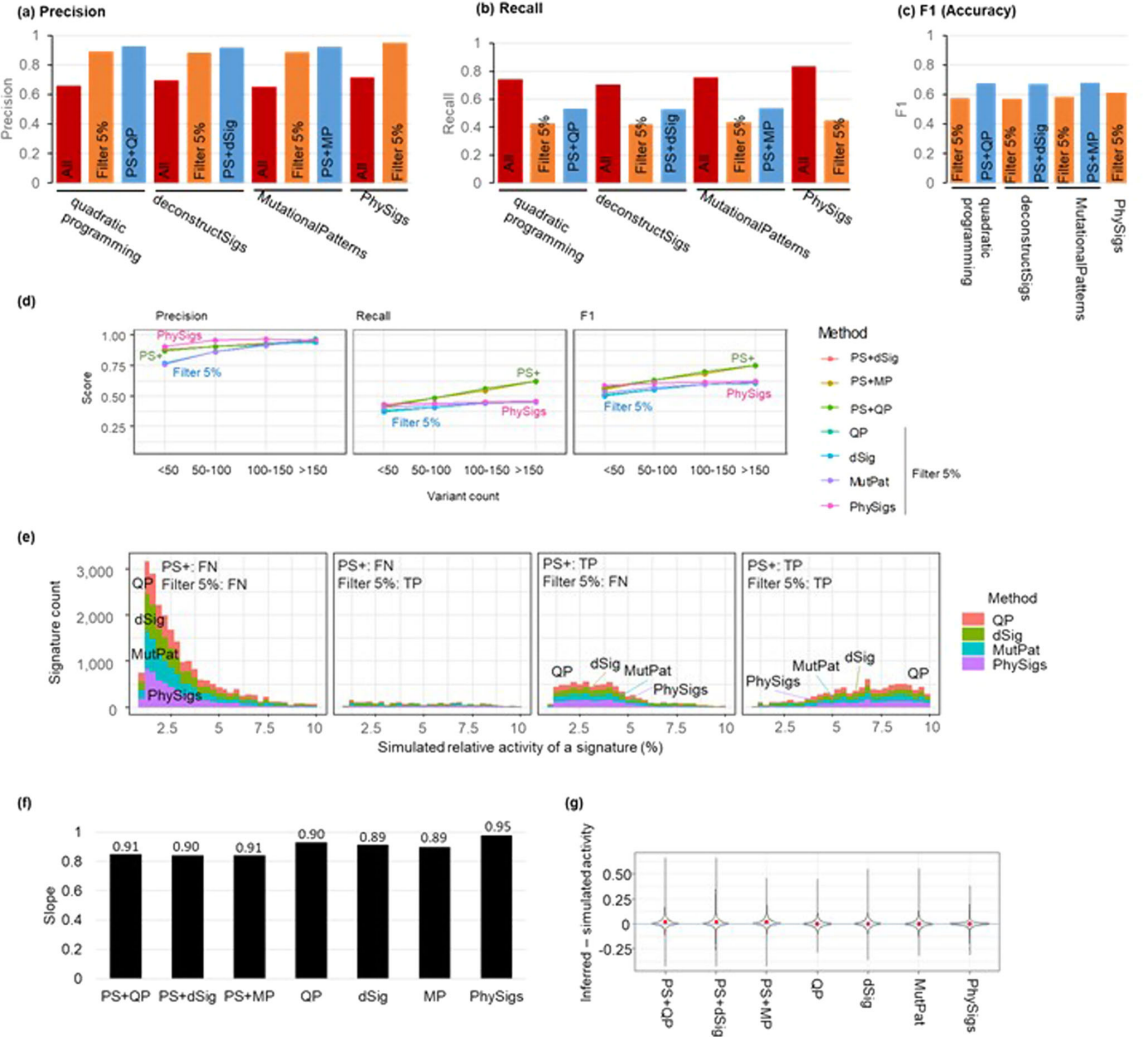
The performance of PhyloSignare. **a** Precision, (**b**) recall, and (**c**) F1 score for all the signatures across all datasets for QP, deconstructSigs (dSig), and MutationalPatterns (MP) without (red) and with *PhyloSignare* (PS) approach (blue, PS + QP, PS + dSig, and PS + MP, respectively), PhySigs, and those with removing signatures with <5% estimated relative activity (orange). Signatures were pooled across all datasets in the computation. Precision was computed as the number of correct signatures detected divided by the total number of signatures detected. The recall was the number of correct signatures detected divided by the total number of simulated signatures. F1 = 2× Precision×Recall/(Precision+Recall). **d** Performance was compared among various numbers of variants per branch. The 5% filtering was applied for all the methods except for PS. **e** Detection of faint signatures (<10% simulated relative activities) was compared between *PhyloSignare* and the other methods (QP, dSig, MutPat, and PhySigs) with filtering out signatures with low inferred relative activities (<5%). **f** Regression slopes between the simulated and inferred activity of signatures. The number above a bar is the *R*^2^ of the statistical fit. **g** The difference between inferred and simulated activities of correctly identified signatures (average marked by red dots).

In the analysis of simulated datasets, we provided all 30 COSMIC v2 signatures for the signature detection because these signatures were randomly selected to generate the simulated datasets. We found that PS + QP produced a much smaller number of incorrect signatures than the direct use of QP. The proportion of correct signatures among detected signatures (precision) was 93% for PS + QP compared to 66% for QP (Fig. 4a). The overall_iS was better for PS + QP than QP (Supplementary Fig. S3b).

PS + QP was also more accurate than simply filtering low-activity signatures (<5%) detected by QP. The simple filtering approach did decrease the false-positive rate of QP, but this improvement came at the expense of higher false-negative rates (worse recall; Fig. 4b). This made the overall performance (F1) of PS + QP better than QP (Fig. 4c). PS + QP removed incorrect signatures more frequently than the 5% filtering for branches with a small number of variants (<50), retaining a similar recall rate to the 5% filtering. On the other hand, PS + QP identified more correct signatures (better recall) for a larger number of mutations (>100) with a similar precision rate to the 5% filtering. This result indicates that the PS system efficiently removes incorrect signatures for a smaller number of variants and can identify more correct signatures for a larger number of variants than the 5% filtering. Similar patterns were observed for PS + dSigs and PS + MP (Fig. 4a–c). Lower and higher filtering cut-offs (1% and 10%, respectively) did not perform as well as 5% cut-off for simple filtering, so the PS approach can offer better accuracy.

A major performance difference between coupling with PS and the 5% filtering is observed to detect signatures with low activity (faint signatures). As expected, the 5% filtering approach cannot detect signatures with activities that are lower than 5%. We found that PS detected more true faint signatures than the 5% filtering (Fig. 4e). Therefore, the application of PS can be useful to detect such faint signatures to certain extent.

### Comparison of PhyloSignare (PS) with a bootstrap approach

Huang, et al.^27^ proposed resampling of mutations to compute variance of estimated relative activities of signatures, which can place confidence limits on each of detected signatures, i.e., percent bootstrap replicates for a given signature detected, with a high value suggesting a robust detection. Therefore, we compared the performance of PS + QP with a bootstrap approach (QP + BS; 1,000 replicates) to filter spurious signatures branch-by-branch. We retained signatures that were detected in >60%, >70%, >80%, >90%, and >95% bootstrap replicates. At higher bootstrap cut-offs, a larger number of incorrect signatures were eliminated, but a larger number of correct signatures were also lost compared to PS + QP (Supplementary Fig. S4a and S4b). Consequently, PS + QP produced a better F1 score than QP + BS (Supplementary Fig. S4c). Therefore, PS may be preferred over the bootstrap approach.

### Comparison of PhyloSignare (PS) with another phylogeny-based method

We further compared the performance of PS + QP with PhySigs that uses the clone phylogeny. PhySigs produced too many false positive signature detections with low activity. So, we applied a 5% filtering to PhySigs results. The rate of incorrect signature detection of PhySigs became similar to PS + QP, but PS + QP produced a slightly better recall rate (Fig. 4a, b). Interestingly, the PS + QP recall rate was better than that of PhySigs for a larger number of variants (>100; Fig. 4d). Also, PS + QP successfully identified more faint signatures correctly than PhySigs (Fig. 4e), while PhySigs produced slightly better relative activities of signatures than PS + QP (Fig. 4f, g). Similar trends were seen for PS + dSig and PS + MP.

### Signature detection for a whole tumor using PhyloSignare (PS)

**Fig. 5:**
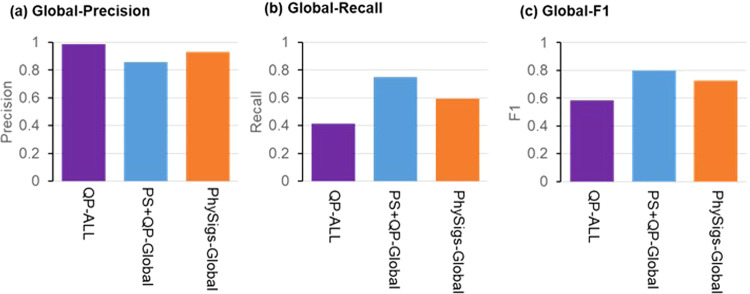
Detection of signatures for a whole tumor. **a** Precision, (**b**) recall, and (**c**) F1 score were calculated for the detection of signatures for a whole tumor (an entire phylogeny). For the QP-ALL approach, variants from all phylogeny branches were pooled, and signatures were estimated. For the PS + QP-Global and PhySigs-Global approaches, signatures were detected for each branch and then were pooled.

Although PS is designed to detect signatures for individual branches, we can also apply it to obtain signatures of a whole tumor (global signatures) by pooling all detected signatures in the phylogeny (PS + QP-Global). We compared how well PS + QP-Global performed compared to the application of QP to the pooled collection of all variants mapped to all branches of the phylogeny (QP-ALL). PS + QP-Global showed higher F1 than QP-ALL, with PhySigs-Global showing an intermediate performance (Fig. 5c). Generally, recall for PS + QP-Global was much better than others (Fig. 5).

### Dynamics of mutational signatures in lung cancer patients

**Fig. 6:**
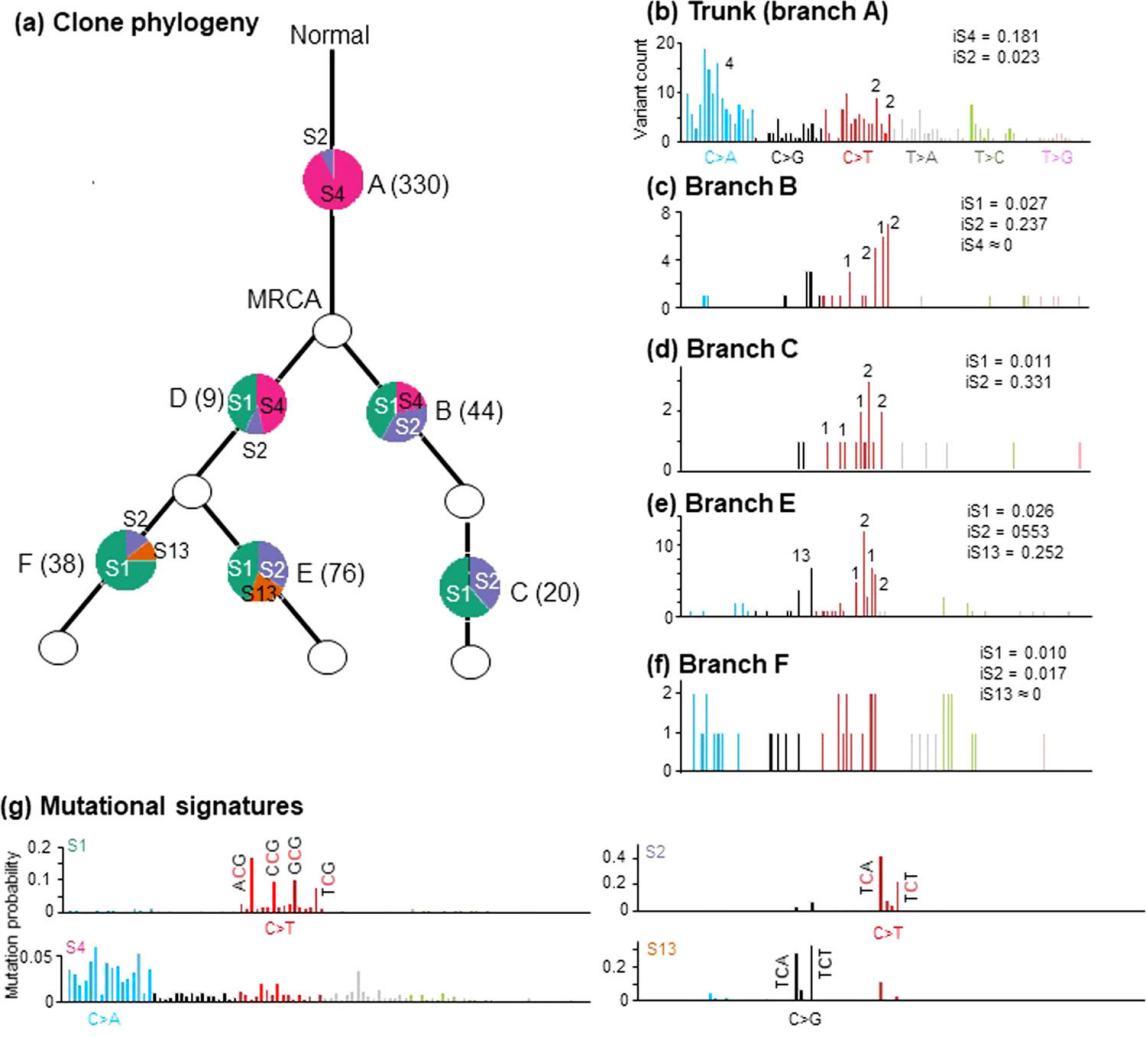
PhyloSignare (PSQP) inferences on CRUK0025 patient data. **a** Clone phylogeny and the mutational signatures identified for different branches (A − F). The number in the parentheses is the variant count for each branch, and a pie chart shows the relative activities of mutational signatures. The most recent common ancestor (MRCA) of all observed clones is marked. **b**–**f** Distribution of variants observed at each branch. The numbers on top of the vertical bars correspond to variant types that were important for COSMIC signatures detected. **g** Distribution of variants for four COSMIC signatures detected for this phylogeny (S1, S2, S4, and S13).

To further test the performance of PS, we next analyzed 61 lung adenocarcinoma clone phylogenies (Supplementary Fig. S1). To perform PS, we provided signatures known to be associated with lung cancer because the other signatures are not expected to be active (see *Methods*). The clone phylogeny of one patient (Fig. 6) consisted of six branches, with branch A (trunk) containing 330 variants and fewer than 100 variants mapped to all other branches. In the trunk, PS predicted the presence of S4—a signature of a smoking-related mutational process that produces many C → A variants (Fig. 6a, g). Indeed, most observed variants were C → A (Fig. 6b). Consequently, S4 received the highest activity estimate (93%) with high iS support (0.18).

COSMIC signature S2 was also active in the trunk, associated with the APOBEC family of cytidine deaminases^20,23^. The activity of S2 was 13 times lower than S4 in the trunk but much higher than S4 in the rest of the branches in the clone phylogeny (Fig. 6a). The activity of S4 was lower in the direct descendants of the most recent common ancestor (MRCA), and it became too small to be detected in the tip branches C, E, and F. Therefore, the mutational processes giving rise to S4 appear inoperative later in tumor evolution (Fig. 6a). Another APOBEC mutational signature, S13, was detected only in tip branches E and F. In comparison, the contribution of S1, the age-related mutational signature, was high in all the branches (Fig. 6a). The only exception was the trunk, probably because the relative activity of S4 was so high that S1 activity was relatively too small to be detected.

The original analysis of this lung adenocarcinoma data also presented mutational signatures for some branches^8^, applying deconstructSigs and then manually selecting at most one mutational signature for each branch. For example, this study reported S4 in the trunk (A) and APOBEC mutational signature in its two descendants (B and E), which PS also identified. No mutational signatures were presented for branches C, D, and F, indicating that coupling available methods with PS enabled signatures to become detectable for these branches.

This dataset was also previously analyzed with PhySigs^32^, which reported many more signatures with appreciable activities for each branch in the phylogeny. As noted earlier, PhySigs tends to produce false positives with relatively low estimated activities. For many branches, simple filtering at a 5% activity level generally produced PhySigs results similar to PS. A major difference from the PS inference is that PhySigs assigned the same signatures (S1, S2, S4, S5, and S6) for three branches (A, D, and F). On the other hand, the branch-by-branch result produced by PS suggested signatures S2 and S4 for branches A, S1, S2, and S4 for D, and S1, S2, and S13 for F. For these branches, we found that signatures detected only by PhySigs were not supported by iS (~0.0), including those with high estimated activities by PhySigs are potentially incorrect (e.g., S4 at F). Overall, PS was able to detect signatures reported in the previous study, validating the performance in empirical data analysis.

**Fig. 7:**
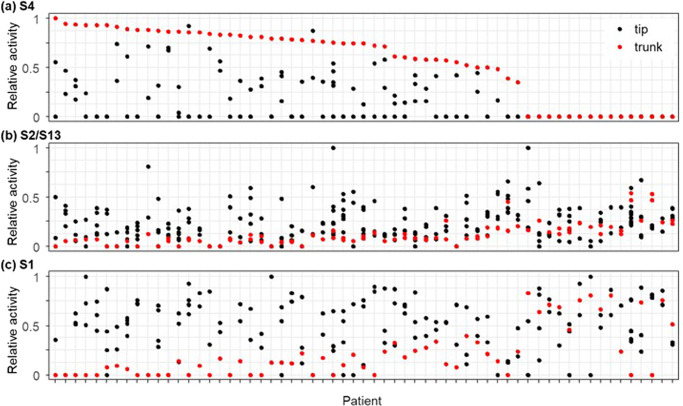
Evolutionary dynamics of mutational signatures. Relative activities of signature S4 (**a**), S2/S13 (**b**), and S1 (**c**) in the trunk (red) and tip (black) branches are shown for each patient. Patients are ordered by the relative activity of S4 in the trunk.

The evolutionary dynamics of mutational patterns for patient CRUK0025 were recapitulated in data analysis from 60 additional patients. S4 had the highest relative activity in the trunk of clone phylogenies of more than 72% of the patients (44/61). Often, S4 activity declined over time, such that it became low in tips compared to the trunk (Fig. 7a). APOBEC mutational signatures (S2 and/or S13) were also active in a vast majority of patients (>86%), with at least one of them found in the trunk branch in most patients (Fig. 7b). Their activity became comparable or higher than S4 in the tips. The age-related S1 signature’s relative activity levels became higher in tips than trunks (Fig. 7c). The diminishing of signature S4 activities and gains of S2/S13 over time that PS demonstrates are consistent with the previous studies^8^, supporting the accuracy of PS. Since the other studies could not resolve branch-level identification of signatures, PS has enabled a higher resolution that identifies clone lineages and branches that have experienced gain and loss (too low to be detected) of dominant signature activities.

**Fig. 8:**
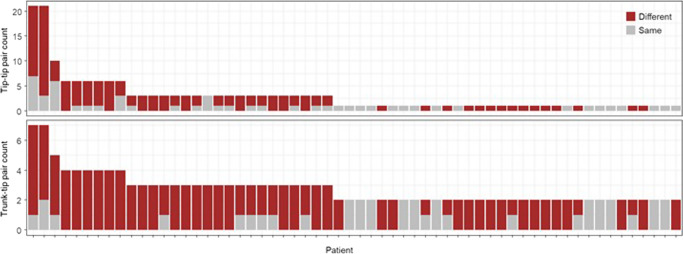
Comparison of signature composition between a pair of branches. Counts of tip-tip branch pairs (top) and trunk-tip pairs (bottom) for each patient. Patients are ordered based on the number of branches in their clone phylogeny. The number of branch pairs containing different (brown) and same (gray) sets of signatures is shown.

For example, a further application of PS directly compares the presence/absence of mutational signatures between a pair of branches within a phylogeny. A comparison between the trunk and tip branches (trunk-tip comparison) can quantify differences between mutational processes active in the earliest and each of the latest branches in patients. We, therefore, constructed 162 trunk-tip comparisons. In a vast majority of pairs, there was a difference for at least one pair of trunk-tip branches (Fig. 8). The main difference was the loss/diminished activity of S4 and the gain of S1 as a dominant signature (Fig. 7). Different sets of dominant mutational processes were operating in the two phases of clonal evolution, which is consistent with suggestions from the previous studies^11,25,33,34^.

In addition, PS’s branch-specific signature detections further identified tip branches that conserved the same composition of mutational signatures as the trunk, while other tip branches within the phylogeny had different signature compositions (Fig. 8). This result indicates that not all new clones differentiate the mutational processes. Such inferences require branch-specific signature prediction, which became possible by PS.

We next tested the differences in signature compositions between the most recently diverged clonal lineages (tip-tip branch pairs). We conducted 176 tip-tip comparisons. More than half of the clone phylogenies had at least one pair of tip-tip branches with different compositions of mutational signatures (Fig. 8). Therefore, as new clones originate, mutational processes may also evolve, increasing the heterogeneity of mutational processes among clone lineages.

## Discussion

*PhyloSignare* can make it possible to detect changing dynamics of mutational processes over time in a patient with high precision. Mutational signature patterns across patients showed convergence towards a loss of smoking-related signatures, consistent with previous lung cancer evolution reports^11^. We also found a convergent tendency to gain APOBEC signatures in MRCA’s descendants, suggesting that mutational processes often shift when the early tumor cells diverge from MRCA over time. There is also a tendency for mutational signatures to diverge among closely related lineages (e.g., tip-tip pairs), suggesting regional and/or temporal differences in tumor mutational and selective pressures.

We did not always detect S1, associated with aging, in the trunk, but S1 was otherwise found in most branches in the phylogeny. S1’s ubiquity is reasonable because the mutational processes due to aging should be present throughout. But, its detection in the presence of S4 seems to be difficult because the probability of C → T mutations (which are the characteristics of S1) under S4 activity is not zero. It means that the high activity of S4 may produce a comparable number of C → T mutations as those induced by S1 activity. Therefore, the much stronger activity of S4 likely overwhelms S1’s signal. The same issue is expected for the detection of some other COSMIC signatures. For example, S6 activities were detected in only a small subset of patients (<30%) in Jamal-Hanjani et al.^8^’s dataset. In this case, distinguishing S6 from S1 and S2 is difficult because they involve C → T mutations. So, some of the absence of the S6 signature could be due to the detection problem, i.e., false negatives. Another lung-related signature, S5, was also not often detected because it is a flat signature (i.e., many different types of mutations occur with a similar probability), whose detection is notoriously difficult even with strong activities^36^. Therefore, the absence of some expected lung cancer signatures does not mean that those mutational processes are inactive. Additional information may help to predict these difficult signatures, e.g., to predict S6 activities, orthogonal evidence of these samples having MMR deficiency.

Identifying lineage-specific mutational signatures has been challenging partly because the number of variants that are needed to make a reliable inference has been rather large^30^. One way to address this problem is to conduct whole-genome sequencing (WGS) to collect hundreds of variants for each branch in the clone phylogeny^14,37^. However, there may not be enough variants per branch even in genome-scale investigations if new clones frequently arise, resulting in short branch lengths, or if somatic evolution has been occurring for a short period or with a slow rate. Also, exome sequencing is currently more commonly used in research investigations, which means that the number of variants mapped to individual branches may not be large enough for existing methods, i.e., their performance is potentially not optimal^30^. Therefore, *PhyloSignare* is likely to be useful to improve the quality of mutational signature identification for individual branches of clone phylogenies in many future investigations.

To optimize the performance of *PhyloSignare*, we suggest using only expected signatures. For example, when we used all 30 COSMIC signatures to analyze a lung cancer patient, CRUK0025 (Fig. 6a), *PhyloSignare* detected a few signatures that were not expected for lung cancer, i.e., potentially spurious signatures (Supplementary Fig. S5a). The detection of these spurious signatures can be easily avoided by providing only expected signatures for a given cancer. This step seems to be especially important when the number of the signature collection is large, e.g., COSMIC v3. For example, using all COSMIC v3 signatures produced a larger number of spurious signatures, while the restriction to the expected signature for the cancer type essentially produced the same results as when the COSMIC v2 signature was used (Supplementary Fig. S5b and S5c).

Another tip for *PhyloSignare* analysis is to be aware of potential underestimation of the number of gain and loss (too low to be detected) of signatures, as neighboring signatures may be incorrectly detected at the final step of *PhyloSignare*. This error happens because *PhyloSignare* assumes the activity of neighboring signatures at a given branch of a phylogeny. Although *PhyloSignare* tests the presence of neighboring signatures, the test may produce an incorrect prediction. This issue is similar to the “signature bleeding” in detecting signatures from cohort data, where signatures present in only some patients are erroneously assigned to the other patients^36^. This error happens because this type of analysis assumes that all patients within a cohort share a similar mutational signature landscape.

Also, in the *PhyloSignare* analysis, a spurious signature loss can happen, especially for “flat or unstable” signatures (e.g., S3, S5, and S8), when two or more flat signatures are among the candidate signatures. In such a case, the iS score can be small for all of them and they maybe incorrectly removed. Since detecting “flat” signatures is known to be difficult by any methods^27^, additional information, e.g., MMR deficiency, may help for the detection. In the future, we plan to advance *PhyloSignare* so that additional information can be jointly used for signature detection.

Last, we analyzed only Single Base Substitution (SBS) Signatures in this study. In addition to SBS signatures, indel and doublet signatures are already available. Technically, these signatures can also be used with *PhyloSignare*, but we will test the accuracy and plan to advance *PhyloSignare* if necessary. In conclusion, *PhyloSignare* can improve the accuracy of mutational signatures detected using standard methods. Its application reveals the dynamics of mutational signatures at a higher phylogenetic resolution, enabling the comparison of mutational activity over time and among closely related lineages.

## Methods

### PhyloSignare (PS) approach

*PhyloSignare* first identifies candidate signatures for each branch by applying a user-selected mutational signature detection method, e.g., quadratic programming (QP) technique^27^, deconstructSigs^28^, or MutationalPatterns^29^ (Fig. 3a). *PhyloSignare* also searches for candidate signatures for a branch by applying the selected mutational signature detection method to (1) each pooled collection of variants from a sibling branch, (2) each pooled collection of variants from the direct ancestral branch, (3) each pooled collection of variants from a direct descendant branch, and (4) a pooled collection of variants from all of these neighboring branches. The objective of pooling information with neighboring branches is to increase the number of variants that enhance existing methods’ statistical power to detect mutational signatures with low activity. Using a signature detection method, we estimate the relative activity of user-given signatures (e.g., COSMIC signatures) in these collections. Mutational signatures with estimated activity greater than 0.01 in at least one collection were included to assemble a set of candidate signatures for a branch. We selected this 0.01 cut-off value because almost half of the incorrect signatures that QP detected had <0.01 estimated relative activities in our simulation study.

We next test the significance of the predicted signature activities. For every candidate signature (*S*), we compute a simple importance score (iS),

1 $${{{{{\rm{iS}}}}}}=\frac{\left({f}_{S-}-f\right)}{f},$$

where,

2 $${f}_{S-}=\sqrt{{{{\sum }}}_{i}{\left({m}_{{iS}}-{o}_{i}\right)}^{2}}.$$

In this equation, *m*_*iS*_ is the estimated count of a mutation type, *i*, when signature *S* is excluded, i.e., the *m*_*iS*_ is obtained by calculating a product of the mutational signature matrices specified, estimated relative activities, and the total mutation count. More specifically, for a candidate signature collection (e.g., *k* signatures), we estimate relative activities for the given branch by using a refitting method (e.g., QP) in which one candidate signature is excluded. That is, we run a refitting method for each candidate signature exclusion. The *o*_*i*_ is the observed count of a mutation type, *i*. Lastly, the summation goes over mutation types, *i*. The other term is,

3 $$f=\sqrt{{{{\sum }}}_{i}{\left({m}_{i}-{o}_{i}\right)}^{2}},$$

where *m*_*i*_ is an estimated mutation count of a mutation type, *i*, when signature *S* is included. Therefore, iS is based on these values with and without exclusion of a candidate signature. iS is expected to be close to zero if a given signature *S* is spurious, i.e., such signatures are unlikely to contribute significantly to the fit of the observed data; we retain signatures with iS > 0.02 (Supplementary Fig. S3a). This iS assessment does not involve optimization of any functions nor calculation of statistical significance because such statistics are often powerless for a limited number of mutations for a branch.

For each branch, the presence of each candidate signature is evaluated by calculating iS, because signatures that are detected only when mutations are pooled with neighboring branches may not be active for a given branch. Similarly, only signatures with iS > 0.02 are retained for a branch.

In the final step, *PhyloSignare* examines the collection of detected signatures for each branch and tests the presence of signatures that are detected only for immediate neighboring branches. Signatures detected for a branch are pooled with those detected only at its immediate relatives. Using the collection of these signatures, their relative activities are estimated with the selected signature detection method. Since signatures that are not present on a branch should not be detected, this step is meant to minimize spurious gain and loss of signatures caused by a small sample size.

In the above, we assumed that the clone phylogeny is known. In empirical data analysis, one needs to generate it using available computational tools for bulk and single-cell sequencing methods; see reviews in the accuracy of methods^7,38,39^. The errors in the collection of variants for each branch (errors in inferred clone phylogeny) will lead to false-negative detection of signatures due to diluted signals caused by incorrect variants and correct variants that are not assigned to a branch. Therefore, we encourage users to scrutinize the quality of inferred clone phylogenies before applying *PhyloSignare*. Also, using different signature collections from the COSMIC v2 collection requires caution, although users are technically allowed to provide COSMIC v3 or their signature collections in *the PhyloSignare* approach. We implemented currently available methods to estimate signature activities (QP, deconstructSigs, and MutationalPatterns) which have been benchmarked only for COSMIC v2 signatures^27–29^.

### Collection and analysis of simulated datasets

We obtained 180 simulated datasets from the website https://github.com/elkebir-group/PhySigs^32^. Each clone phylogeny (containing five or seven branches) can be partitioned into up to three subtrees, each with an identical set of mutational signatures and relative activities. Each branch of these clone phylogenies had from 2 to 205 mutations. COSMIC v2 signatures were randomly sampled to select a set of signatures for each branch of a phylogeny. Relative exposures of selected signatures at each branch were determined by drawing from a symmetric Dirichlet distribution. Observed mutation counts at each branch were generated by introducing Gaussian noise with a mean of zero and standard deviation of 0.1, 0.2, or 0.3.

The phylogeny of the dataset shown in Fig. 2a was modeled after CRU0079 data^8^. Each branch experienced 20–100 mutations caused by three mutational processes. Branch A harbors signatures S17, whereas the descendant branches (B and C) have one new mutational signature (S13). Further clonal evolution depicted in branches D and E acquired a new mutational signature (S1). Using an available software to generate mutation counts^40^, the relative activity of each signature for a branch was assigned by drawing from a Dirichlet distribution, and observed mutation counts were generated from a multinomial distribution.

We applied *PhyloSignare* to these simulated datasets by providing correct clone phylogenies and COSMIC v2 signatures obtained from https://cancer.sanger.ac.uk/cosmic/signatures. For each branch mutation count, we also performed QP^27^, deconstructSigs^28^, and MutationalPatterns^29^ by providing COSMIC v2 signatures. Here, signatures that were estimated with <0.001 relative frequencies were considered to be absent. deconstructSigs was performed by using the option to discard inferred signatures with <0.001 relative frequencies. We did not use deconstructSigs’ function to normalize variant counts because the uniform distribution of variants was assumed in the simulation. The bootstrap option in QP was performed by generating 1,000 bootstrap replicate datasets for each branch. We excluded branches with <20 variants from the accuracy evaluation because signature detection is impossible for any method. PhySigs inferences were obtained from https://github.com/elkebir-group/PhySigs, and the software was downloaded in 2019.

We also used CloneSig (downloaded in 2021) to analyze the dataset generated using the phylogeny in Fig. 2a. Since CloneSig assumes a linear phylogeny, we separately analyzed each section of the phylogeny, i.e., branches A and B, branches A, C, and D, and branches A, C, and E. Also, CloneSig requires an observed read count for each mutation. We assigned 2,000 for wild-type variants for all the mutations, and the mutant-type variant count was set to 1000, 900, and 800 for variants from branch A, branches B and C, and branches D and E, respectively. CloneSig analysis was performed assuming the tumor purity equal to 1.

### Collection and analysis of empirical datasets

We obtained 100 non-small cell lung cancer (NSCLC) data from the TRACERx Lung Cancer study^8^. We collected only invasive adenocarcinoma and squamous cell carcinoma samples (61 and 32 samples, respectively) because the number of the other cancer types was very small. These datasets contained inferred clone phylogenies with all observed mutations mapped along branches. We selected the primary phylogenies when more than one phylogeny was reported. We then excluded datasets when the total number of variants was less than 100 or when a clone phylogeny did not have at least two tip branches. After these filtering steps, we obtained clone phylogenies from 61 patients.

We classified each observed mutation into the 96 trinucleotide mutation patterns and generated branch-specific mutation counts used as input information for *PhyloSignare*. When a mutation count for a branch was < 20, we pooled them with its neighboring branch because it was impossible to identify mutational signatures on data with a too-small number of mutations (red branches in Fig. 2). To perform the *PhyloSignare* analysis, we used COSMIC v2 signatures known in lung adenocarcinoma (S1, S2, S4, S5, S6, S13, and S17) and squamous cell carcinoma (S1, S2, S4, S5, S13). Accordingly, we provided each set of known signatures in the analysis based on the given dataset’s cancer type. For COSMIC v3 signatures, we used those for lung cancer (SBS1, SBS2, SBS3, SBS4, SBS5, SBS6, SBS9, SBS13, SBS15, SBS17a, SBS17b, SBS18, SBS28, SBS29, SBS40). We used QP to estimate relative activities in all our data analyses.

### Reporting summary

Further information on research design is available in the Nature Research Reporting Summary linked to this article.

## Supplementary information

Supplementary information

Supplementary Data 1

Supplementary Data 2

Supplementary Data 3

Supplementary Data 4

Reporting Summary

Description of Additional Supplementary Files

## Data Availability

No new simulated or empirical genetic variation datasets were generated for this study. We used existing datasets obtained from Supplementary material of ref. ^8^ and https://github.com/elkebir-group/PhySigs^32,41^. These data were converted into input files for use in PhyloSignare that can be downloaded from https://github.com/SayakaMiura/PhyloSignare/input_files^42^. The source data for figures are available at https://github.com/SayakaMiura/PhyloSignare/Sourcedata^42^ and in Supplementary Data 1-4.

## Code availability

The *PhyloSignare* code is available at https://github.com/SayakaMiura/PhyloSignare^42^.
