# Supplementary material for: A phylogenetic approach to study the evolution of somatic mutational processes in cancer
Source: Commun Biol. 2022 Jun 22;5:617. doi: 10.1038/s42003-022-03560-0 (PMC9217972; doi:10.1038/s42003-022-03560-0)
Supplement: Supplementary file 1 — Supplementary information [file 42003_2022_3560_MOESM1_ESM.pdf]

(a)

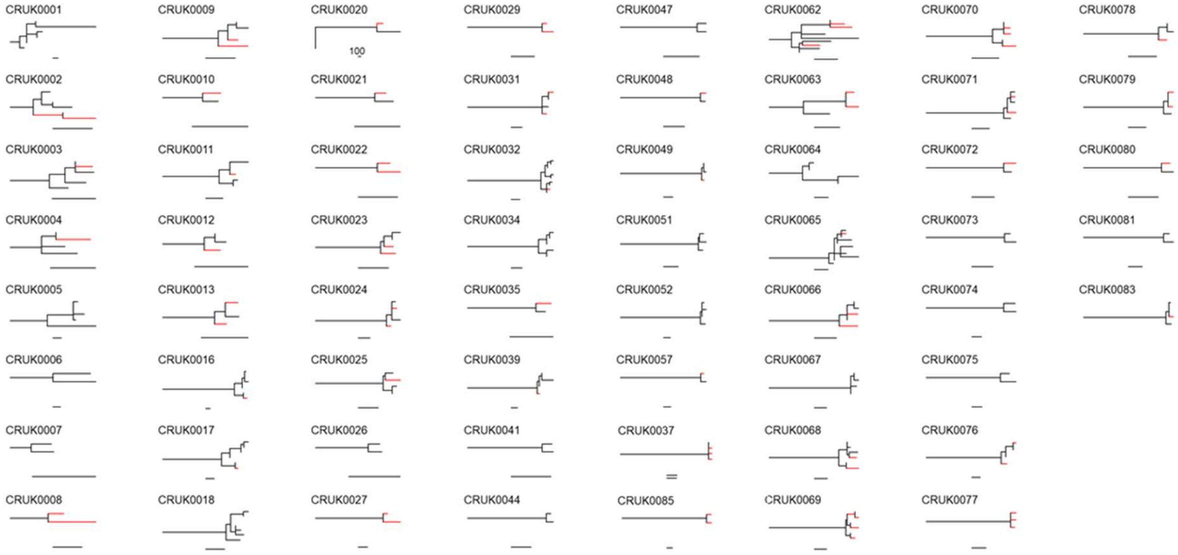

(b)

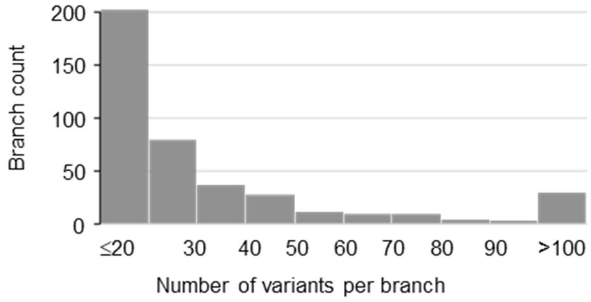

Figure S1. Clone phylogenies from data in Jamal-Hanjani et al. (2017). (**a**) Only phylogenies with >1 tip were included. Branches with <20 variants were combined for signature detection (see **Methods**). Combined branches were shown with red. The scale bar is equal to 100 variants in each case. (**b**) The number of variants in individual branches of all the clone phylogenies that are shown in panel **a**.

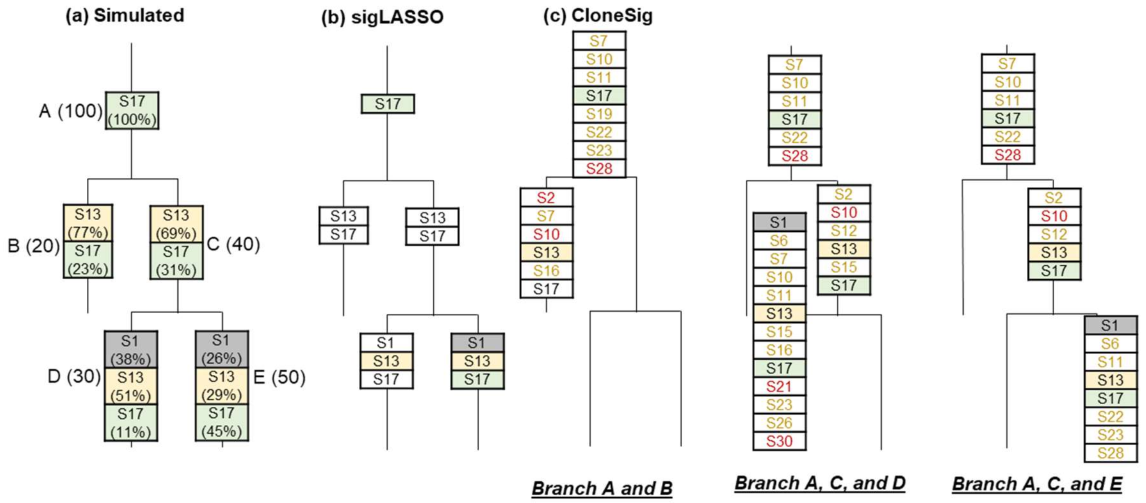

Figure S2. **Mutational signatures detected by sigLASSO and CloneSig**. (**a**) Model clone phylogeny and simulated mutational signatures (see **Figure 2a** for the detail). (**b** and **c**) mutational signatures inferred by sigLASSO (**b**) and CloneSigs (**c**). (**c**) Since CloneSigs can be used only for a linear tree, we independently analyzed mutations from branches A and B, branches A, C, and D, and branches A, C, and E. Incorrectly detected signatures are shown with red (>5% estimated relative activity) and yellow (0.1% - 5% estimated relative activity) letters, and correct signatures not detected are shown in white boxes with black letters.

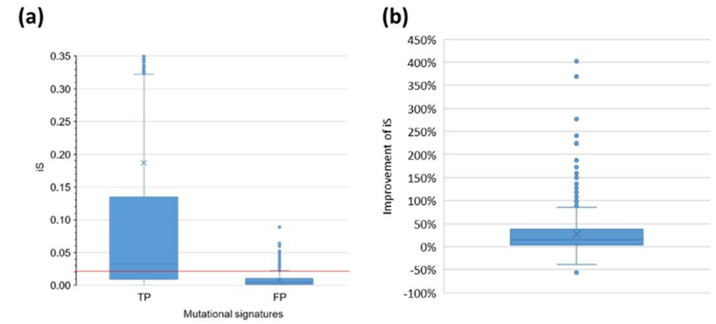

Figure S3. The pattern of iS values. (**a**) iS values for correct (TP) and incorrect (FP) signatures. The red line indicates iS of 0.02. (**b**) Difference of iS before and after applying *PhyloSignare*. We summed up iS values from all signatures detected from each branch of a phylogeny (overall iS). We computed the overall iS for each phylogeny and compared the values between before and after applying *PhyloSignare*. For each phylogeny, we calculated the difference of overall iS after applying *Phylosignare* from the value before applying *PhyloSignare*, which was then divided by the value before applying *PhyloSignare* to normalize it. QP was used for the signature detection, and the iS was calculated for these signatures. All the simulated datasets were used (see **Methods**). A cross in a box is a mean and a center line is a median. A box represents the range between first and third quartile. The whiskers indicate 1.5× interquartile range box limits. The outliers are represented by dots.

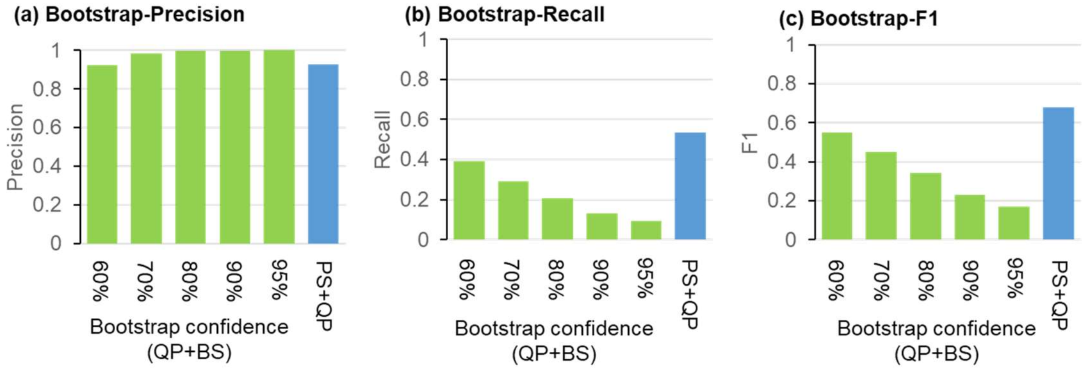

Figure S4: Comparison between *PhyloSignare* and bootstrapping approaches. (**a**) Precision, (**b**) recall, and (**c**) F1 score for all the signatures across all datasets for the bootstrap approach with QP [QP+BS] and QP coupled with *PhyloSignare* (PS+QP). For the bootstrapping approach, signatures that were detected with 60%, 70%, 80%, 90%, and 95% bootstrap confidence are shown. Signatures were pooled across all datasets in the computation.

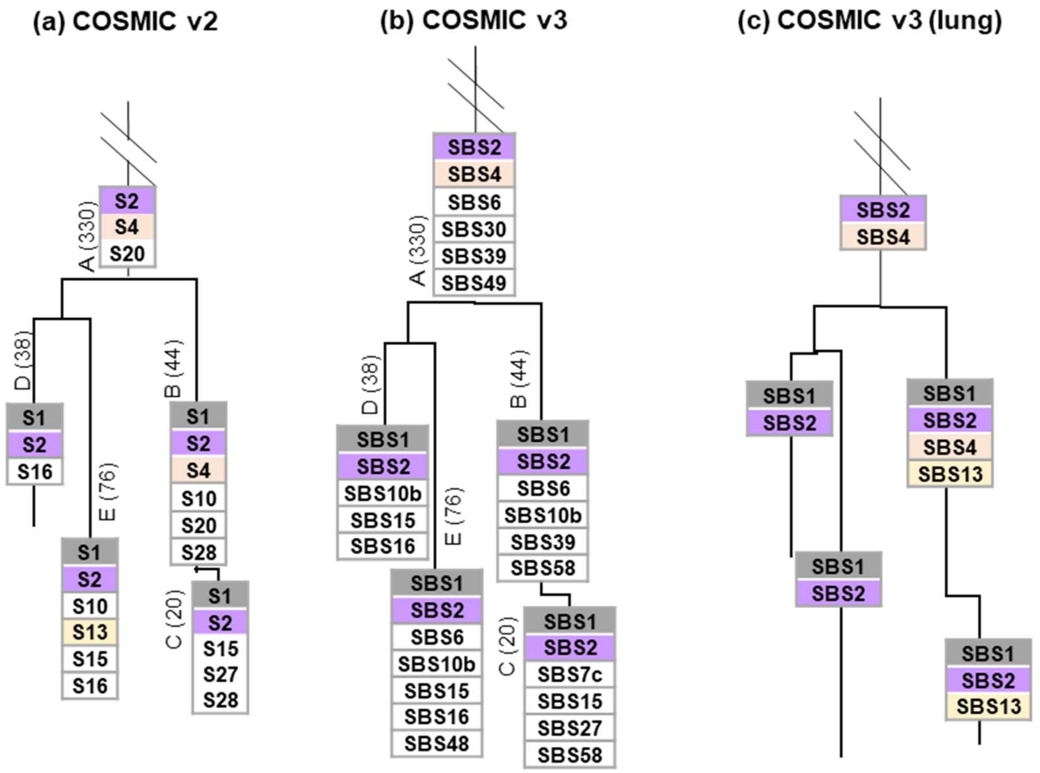

Figure S5. *PhyloSignar*e inferences on CRUK0025 patient data. The mutational signatures identified for different branches (A − F) are shown. The number in the parentheses is the variant count for each branch. QP was coupled with *PhyloSignare*. (**a**) All COSMIC v2 signatures were used. (**b**) All COSMIC v3 signatures were used. (**c**) COSMIC v3 signatures that are known to be found in lung cancer were used. Detected signatures that are not expected for lung cancer are shown within white boxes.
