# Supplementary material for: A phylogenetic approach to study the evolution of somatic mutational processes in cancer
Source: Commun Biol. 2022 Jun 22;5:617. doi: 10.1038/s42003-022-03560-0 (PMC9217972; doi:10.1038/s42003-022-03560-0)
Supplement: Supplementary file 7 — Description of Additional Supplementary Files [file 42003_2022_3560_MOESM7_ESM.pdf]

Description of Additional Information file

File name: Supplementary Data 1-4

Description: Source data for figures 1-8
